# Low-complexity microbiota in the duodenum of children with newly diagnosed ulcerative colitis

**DOI:** 10.1371/journal.pone.0186178

**Published:** 2017-10-19

**Authors:** Fei Sjöberg, Cecilia Barkman, Intawat Nookaew, Sofia Östman, Ingegerd Adlerberth, Robert Saalman, Agnes E. Wold

**Affiliations:** 1 Department of Infectious Diseases, Institute of Biomedicine, University of Gothenburg, Gothenburg, Sweden; 2 Department of Biology and Biological Engineering, Chalmers University of Technology, Gothenburg, Sweden; 3 Department of Biomedical Informatics, College of Medicine, University of Arkansas for Medical Sciences, Little Rock, Arkansas, United States of America; 4 Department of Paediatrics, Institute of Clinical Science, University of Gothenburg, Gothenburg, Sweden; "INSERM", FRANCE

## Abstract

**Background:**

Inflammatory bowel disease (IBD) is characterized by gut dysbiosis. To date, the large bowel microbiota has been in focus. However, the microbiota of the small intestine may also be of importance, as the small bowel is a site for the induction and control of mucosal immune responses, which can be modulated by constituents of the local microbiota.

**Methods:**

Duodenal fluids were collected during diagnostic work-up of treatment-naïve children who were suspected of having IBD. The duodenal fluids were analyzed by pyrosequencing (average of 32,000 reads/sample, read length of 500 nucleotides). After diagnosis, the duodenal microbiota of subjects with ulcerative colitis (N = 8) or Crohn’s disease (N = 5), and non-IBD controls (N = 8) were compared.

**Results:**

Pyrosequencing revealed that the duodenal microbiota of children with ulcerative colitis contained fewer Operational Taxonomic Units (OTUs) per individual than the duodenal microbiota of the controls (*P* = 0.005). This reduction in richness of the duodenal microbiota was seen for three major phyla: *Firmicutes*, *Actinobacteria*, and *Bacteroidetes*. Several bacterial genera were detected less frequently in the children with ulcerative colitis than in the non-IBD controls, including *Collinsella* (*P* = 0.001), *Lactobacillus* (*P* = 0.007), and *Bacillus* (*P* = 0.007), as well as a non-identified member of the order *Sphingobacteriales* (*P* = 0.007).

**Conclusions:**

In this pilot study, we show that the duodenal microbiota of children with ulcerative colitis exhibits reduced overall richness, despite the fact that the inflammation is primarily localized to the colon. These results should be corroborated in a larger study.

## Introduction

Inflammatory bowel disease (IBD) is characterized by chronic inflammation of the gastrointestinal tract. The two major subcategories of IBD are ulcerative colitis and Crohn’s disease. In ulcerative colitis, the colonic mucosa is the main affected site, whereas in Crohn´s disease any part of the alimentary tract can be affected and the inflammation is often transmural with fibrosis and fistula formation [1]. While the cause of IBD is unknown, the underlying mechanism is thought to involve interactions between host genetic factors, a dysbalanced microbiota, and exaggerated immune responses to gut luminal antigens of bacterial origin [2, 3].

Up to 20% of all IBD cases present during childhood [4]. The incidence of childhood-onset IBD has increased over the last few decades in countries that have a Western lifestyle [5, 6]. Compared to adults, children with IBD show more extensive inflammation at the onset of the disease, *e*.*g*., children with ulcerative colitis often present with pancolitis [7, 8]. Furthermore, in Crohn´s disease presenting in childhood, colon involvement is commonly observed [9]. Towards uncovering the etiology of IBD, cases with onset during childhood may be especially interesting to study, as the disease has not yet been long-established and, in general, children have fewer co-existing disorders and, consequently, have received fewer medications than adults.

Several studies have reported that the microbiota of the large bowel is altered in children and adults with IBD [10–13]. In addition, microbiota dysbiosis in the terminal ileum has been reported in pediatric patients with Crohn’s disease [14, 15].

Our hypothesis is that alterations not only to the colonic and ileal microbiota, but also to the proximal small intestinal microbiota play a significant role in IBD. The small intestine is the inductive site for mucosal and systemic immunity to intestinal microbes and is also crucial for the development of oral tolerance, i.e., active immune suppression towards harmless non-microbial proteins [16]. Thus, the small intestinal microbiota may shape both the mucosal and systemic immune systems [17].

The proximal intestinal microbiota differs profoundly in composition from that of the ileum and colon, in that the *Firmicutes* and *Proteobacteria* phyla are dominant, with *Streptococcus* and *Neisseria* being the most prevalent genera, followed by *Veillonella* and *Gemella* [18, 19]. Obligate anaerobes that are commonly found in the large bowel, e.g., the *Bacteroidetes* phylum and Clostridial clusters IV and XIVa belonging to the *Firmicutes* phylum, are found in lower numbers in the small bowel.

We hypothesized that the intestinal microbiota of patients with IBD is altered, not only in the colon and terminal ileum, but also in the proximal part of the small intestine. Here, we characterize the duodenal microbiota of children with newly diagnosed IBD, before the onset of treatment, and compare these microbiota with those of non-IBD controls who report similar intestinal symptoms.

## Patients and methods

### Patients

Children who had clinical symptoms that raised a suspicion of IBD and who were referred to the Sahlgrenska University Hospital in Gothenburg, Sweden, were eligible for inclusion in the study, provided that they were treatment-naïve, i.e., had not been administered antibiotics or anti-inflammatory drugs or undergone any symptom-reducing therapy (e.g., diet restriction, acid suppressants) in the previous 3 months. The diagnostic work-up included esophagogastroduodenoscopy, ileocolonoscopy, and small intestine imaging according to the Porto criteria [1]. In total, 13 children were diagnosed with IBD, including 8 children with ulcerative colitis (5 males and 3 females; median age, 15 years; age range, 9–16 years) and 5 children with Crohn’s disease (2 males and 3 females; median age, 11 years; age range, 9–16 years). IBD was excluded in 8 children (5 males and 3 females; median age, 15 years; age range, 13–16 years) after the diagnostic work-up, as endoscopic examination of these patients revealed neither macroscopic nor microscopic signs of inflammation. These children served as the symptomatic non-IBD controls and include 6 children with “irritable bowel syndrome (IBS) with predominant diarrhea” and 2 patients with “functional diarrhea” according to current classification of “functional bowel disorders” [20].

Ethics statement: Informed consent was obtained from the parents and assent was obtained from the children. The Medical Ethics Committee of the University of Gothenburg approved the study (permit no. Ö192-00).

### Paris classification of the disease phenotype

The disease distribution and behavior of IBD were characterized according to the Paris phenotype classification scheme [9]. All eight children with ulcerative colitis had pancolitis (E4) but lacked macroscopic or microscopic signs of inflammation in the upper gastrointestinal tract. All five cases of Crohn’s disease showed an inflammatory disease behavior (B1) that was non-stricturing and non-penetrating, and all had colonic involvement, i.e., the inflammation was localized to the colon (L2) in one child and to the ileum and colon (L3) in four children. Of the L3 cases, two also had involvement of the upper gastrointestinal tract, i.e., proximal to ligamentum of Treitz (L4a).

### Duodenal fluid collection

The large bowel was cleansed using polyethylene glycol (Laxabon^®^) the day before endoscopy of the small and large intestines. Duodenal fluid was aspirated *via* a sterile tube before the biopsies were sampled using the working channel of the gastroscope; no wash step was used in the fluid sampling procedure. All samples were immediately placed in a sterile gas-tight sachet (AnaeroGen Compact, Oxoid Ltd, Basingstoke, UK) in which an anaerobic milieu was created using an anaerobic generator (Benex Ltd., Shannon, County Clare, Ireland) [21]. Anaerobiosis was confirmed upon arrival to the laboratory by checking the anaerobic indicator included in the sachet. The fluids were stored frozen at -80°C within 24 hours after sampling.

### Pyrosequencing analysis

Bacterial DNA was extracted from 200 μL of duodenal fluid according to the manufacturer’s protocol (QIAamp DNA Stool Mini Kit; Qiagen, Hilden, Germany). To increase the DNA yield [22], four glass beads (3.0-mm diameter) and 0.5 g of zirconia beads (0.1-mm diameter) were added to the ASL buffer and the suspension was homogenized twice at 5 m/s for 30 s (FastPrep Cell disrupter; Thermo Savant, Holbrook, NY, USA), incubated at 95°C for 5 min, and then shaken at 1,200 rpm (Vibrax Shaker; IKA, Staufen, Germany) for 30 min at 5°C. The DNA was quantified spectrophotometrically (NanoDrop Technologies, Wilmington, DE, USA).

#### PCR and DNA sequencing

The V1–V3 region of the bacterial 16S rRNA genes was amplified using: forward primer 27F, 5′-AGAGTTTGATCCTGGCTCAG-3′; and reverse primer 534R, 5′-ATTACCGCGGCTGCTGG-3′. The following PCR protocol was followed: 98°C for 30 s, followed by 25 cycles of 98°C for 10 s, 56°C for 30 s, 72°C for 10 s, and a final extension step at 72°C for 1 min. The PCR products were column-purified and a second PCR was performed to add sequencing adaptors and multiplex identifiers using the same conditions, except that steps 2–4 were performed for only 5 cycles. The products were column-purified again, quantified (Qubit Fluorometer; Life Technologies, Carlsbad, CA, USA), pooled, and gel-purified and the DNA was recovered with a standard PCR gel purification kit. The library was constructed according to the protocol of GATC Biotech (Konstanz, Germany) and sequenced using the Genome Sequencer FLX System (Roche, Basel, Switzerland).

#### Sequence analysis

The raw sequences were assigned to samples according to their barcodes and analyzed using the Quantitative Insights into Microbial Ecology (QIIME) software package, employing default parameters for each step [23], except where specified. Singletons and potential chimeric sequences were removed, and only high-quality reads with a quality score ≥25 and a read length of 200–500 nucleotides (nt) were retained for analysis. Operational Taxonomic Units (OTUs) were chosen based on repetitive sequences derived from sequence clustering using the UCLUST software [24], with a minimum pairwise identity of 97%. A representative sequence for each OTU was chosen based on abundance and length, aligned using the PyNAST software (QIIME), and assigned to the genus level (when possible) using the Ribosomal Database Project (RDP) classifier nomenclature.

### Statistical analysis

Orthogonal Partial Least Squares (OPLS, SIMCA P+ V13; Umetrics AB, Umeå, Sweden) [25] was used to compare the overall patterns of the duodenal fluid microbiota between the three diagnostic groups: ulcerative colitis; Crohn’s disease; and controls. OPLS is a regression variety of principal component analysis that relates the X dataset (the presence or absence of each OTU) to a Y-variable; in this case, the diagnosis. To make the data more easily illustrated, OTUs that contributed little to the model were eliminated after the initial OPLS analysis using the Variable Influence on Projection (VIP) approach [26], applying a threshold VIP-value of 1.5. Univariate analyses were performed using Fisher’s exact tests (SPSS software; IBM Corp., New York, NY, USA) to confirm unequal distribution between the diagnostic groups of those OTUs that showed the strongest contributions in the respective OPLS models. One-way ANOVA was performed to compare overall richness at the phylum and genus levels between the diagnostic groups (Prism 5 software; GraphPad Inc., La Jolla, CA, USA).

## Results

The duodenal microbiota of children with suspected IBD were analyzed, before diagnosis and treatment. After a diagnosis was obtained, the microbial compositions were compared between children with ulcerative colitis (N = 8) or Crohn’s disease (N = 5), and non-IBD controls (N = 8) who had symptoms suggestive of IBD but had neither macroscopic nor microscopic signs of intestinal inflammation.

### Distribution of phyla and unclassified sequences

In all, close to 700,000 high-quality sequences, with a mean (±SD) read length of 495 (±24) nt were obtained, and 322 OTUs were identified using a 97% pairwise identity cut-off (S1 Table).

**Fig 1:**
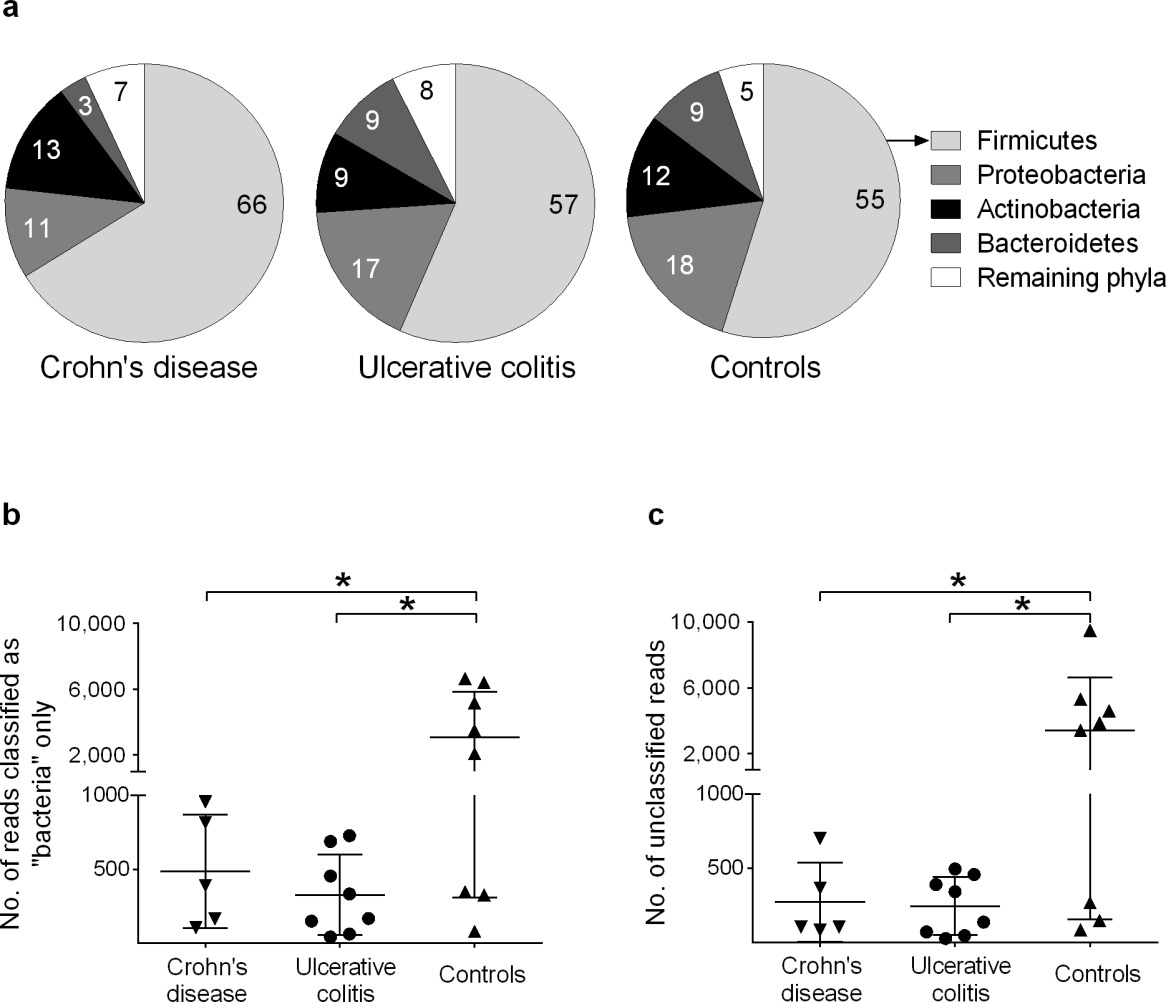
Distributions of reads assigned to phyla and non-classified reads in the duodenal microbiota of children with IBD. DNA was extracted from the duodenal fluid samples and subjected to an analysis of the bacterial 16S rRNA genes (V1–V3 region, with an average read length of 495 nt) using pyrosequencing. **A)** Composition of the microbiota at the phylum level. Four phyla predominate, with no significant differences in distribution between the groups (one-way ANOVA). The “remaining phyla” comprise the 15 phyla listed in the S2 Table. **B)** Numbers of reads that belong to the domain Bacteria but that could not be classified to the phylum level. Each patient is represented by one symbol. **C)** Number of reads per individual that could not be classified as belonging to the domain Bacteria. Error bars denote mean ±1 SD; *, *P*<0.05 (one-way ANOVA).

Altogether, 19 bacterial phyla were represented in the dataset. The phylum distribution pattern did not differ between the three diagnostic groups (Fig 1A). *Firmicutes* comprised 59% of the total reads. Three other phyla were detected relatively frequently: *Proteobacteria* (15%); *Actinobacteria* (11%); and *Bacteroidetes* (7%). The remaining 15 phyla collectively comprised 7% of the reads (S2 Table).

Some reads could be classified as “Bacteria” but could not be assigned to any known phylum. Such sequences were more prevalent in the samples obtained from the non-IBD controls (3.5% of the reads) than in those individuals with either ulcerative colitis (0.4%, *P* = 0.01) or Crohn’s disease (0.4%, *P* = 0.03) (Fig 1B).

Another set of sequences could not even be classified as Bacteria. These reads were also found more commonly in the control children (3.9% of the reads) than in those with ulcerative colitis (0.3%, *P* = 0.01) or Crohn’s disease (0.2%, *P* = 0.03) (Fig 1C).

Pearson’s correlation analyses were performed to determine if the numbers of reads designated as “bacteria only” and “unclassified reads” correlated with each other. The two groups were highly correlated (S1 and S2 Tables), in the patients with Crohn’s disease (r = 0.915, *P* = 0.029; S1A Fig), in the patients with ulcerative colitis (r = 0.915, *P* = 0.029; S1B Fig), and in the controls (r = 0.866, *P* = 0.005; S1C Fig).

### Microbial richness at the OTU level

**Fig 2:**
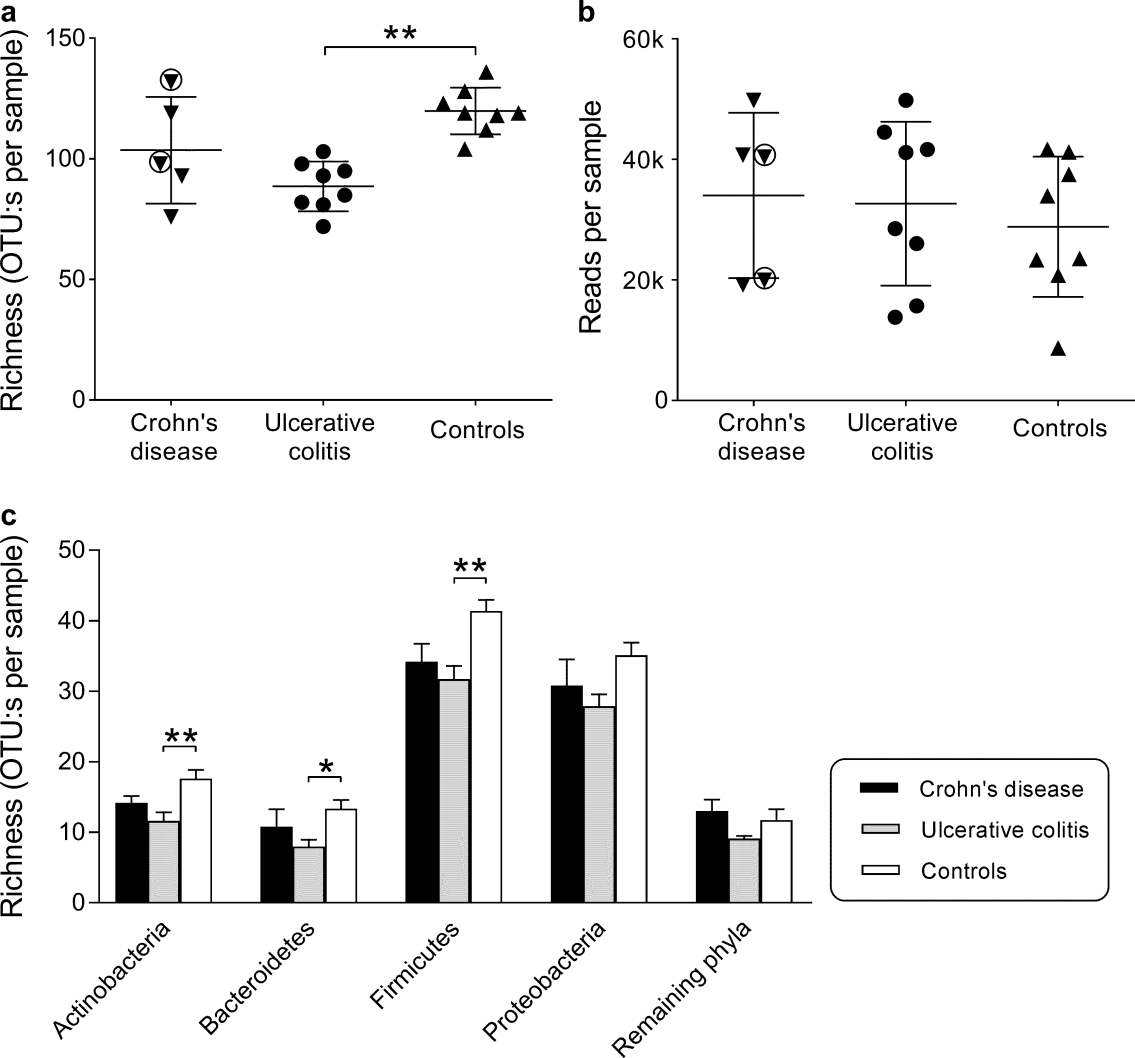
Duodenal microbiota richness in children with IBD and in controls. Bacterial DNA was extracted from duodenal fluid samples and the 16S rRNA genes were pyrosequenced and assigned to unique OTUs (operational taxonomic units), using a minimum pairwise identity of 97%. **A)** Number of OTUs per sample. Each symbol represents one individual. The encircled symbols denote patients with inflammation that affected the duodenum. Values shown are mean ±1 SD; **, *P*<0.01 (one-way ANOVA). **B)** Number of sequence reads per sample. There are no significant differences between the diagnostic groups (one-way ANOVA). **C)** Average numbers of OTUs belonging to each of the four major phyla, and the remaining 15 phyla, in children with IBD and in the controls. Error bars denote the standard error of the mean. *, *P*<0.05; **, *P*<0.01 (one-way ANOVA).

To assess microbial richness, the numbers of OTUs per patient were calculated (Fig 2A). Duodenal fluids from the patients with ulcerative colitis yielded fewer OTUs on average, as compared to samples from the controls (90±10 *vs*. 120±10 OTUs, *P* = 0.005). Samples from patients with Crohn’s disease showed levels of richness that ranged from low to as high as the non-IBD controls with no significant difference noted between these children and the non-IBD controls (Fig 2A).

A low number of OTUs might be a consequence of a low total bacterial population. However, the number of reads per sample, i.e., the amount of bacterial DNA, was not lower for the children with IBD than for the controls (Fig 2B).

We investigated whether the limited microbial richness seen in children with IBD was restricted to certain phyla or occurred across phyla. As illustrated in Fig 2C, the picture was consistent across phyla, with the lowest numbers of OTUs being detected in the duodenal fluids of children with ulcerative colitis and the highest numbers being seen in the duodenal fluids of the non-IBD controls. Although the children with Crohn’s disease tended to have fewer OTUs than the controls across the four major phyla, the differences were not statistically significant.

We also calculated the relative abundance of taxa using Shannon’s index, but found no significant differences between the three groups (data not shown).

### Distribution of individual OTUs in children with IBD and controls

We attempted to classify each OTU found in the dataset (n = 322) to the genus level, which was possible in 232 (72%) of the cases. *Streptococcus* and *Veillonella* were the most prevalent genera, represented by 31% and 12% of the total reads, respectively, with both belonging to the phylum *Firmicutes* (Table 1). In addition, 59 OTUs (18%) could only be assigned to a family, 17 to an order, 11 to a class, and 3 to a phylum.

**Table 1:** Most prevalent taxa.

			Percentage of each sample, mean (±1SD)		
Taxon	Phylum	All samples^a^	Crohn's disease	Ulcerative colitis	Controls
*Streptococcus*	Firmicutes	31	35 (±24)	30 (±12)	21 (±14)
*Veillonella*	Firmicutes	12	8.1(±7.0)	12 (±3.5)	11 (±8.4)
*Haemophilus*	Proteobacteria	4.8	0.32 (±0.20)	8.3 (±9.7)	1.5 (±1.6)
*Prevotella*	Bacteroidetes	4.8	1.2 (±0.83)	7.4 (±10)	5.9 (±5.3)
*Actinomyces*	Actinobacteria	4.3	8.2 (±5.5)	3.8 (±4.2)	3.2 (±2.7)
*Granulicatella*	Firmicutes	3.7	4.6 (±1.8)	4.2 (±2.7)	3.0 (±2.2)
*Neisseria*	Proteobacteria	3.6	1.1 (±1.0)	4.9 (±5.3)	2.8 (±2.7)
*Rothia*	Actinobacteria	3.1	1.6 (±0.84)	4.0 (±4.6)	2.1 (±2.0)
TM7*	TM7	3.0	5.4 (±3.6)	3.4 (±2.9)	1.8 (±1.3)
*Enhydrobacter*	Proteobacteria	3.0	0.04 (±0.05)	0.01 (±0.01)	6.0 (±17)
*Ralstonia*	Proteobacteria	2.6	6.1 (±5.8)	1.8 (±1.2)	1.4 (±0.91)
*Gemella*	Firmicutes	2.1	3.5 (±1.2)	2.1 (±1.3)	1.2 (±0.64)
*Fusobacterium*	Fusobacteria	1.6	0.43 (±0.41)	3.4 (±4.5)	0.81 (±1.3)
*Parvimonas*	Firmicutes	1.2	1.9 (±1.2)	1.4 (±1.4)	0.42 (±0.60)
*Micrococcus*	Actinobacteria	1.1	0.17 (±0.18)	0.01 (±0.02)	2.0 (±5.7)
*Megasphaera*	Firmicutes	1.0	1.7 (±1.7)	0.88 (±0.48)	0.87 (±0.84)
*Oribacterium*	Firmicutes	0.96	1.1 (±0.77)	0.97 (±0.41)	0.76 (±0.70)
*Solobacterium*	Firmicutes	0.94	1.3 (±1.1)	0.90 (±0.48)	0.81 (±0.59)
*Lachnospiraceae*	Firmicutes	0.71	1.4 (±1.1)	0.56 (±0.26)	0.43 (±0.31)
*Leptotrichia*	Fusobacteria	0.69	0.30 (±0.32)	0.35 (±0.20)	1.4 (±1.7)
*Porphyromonas*	Bacteroidetes	0.66	0.62 (±0.76)	1.0 (±1.4)	0.66 (±0.64)
*Propionibacterium*	Actinobacteria	0.64	1.4 (±0.77)	0.24 (±0.35)	0.77 (±0.95)
*Atopobium*	Actinobacteria	0.61	0.48 (±0.39)	0.77 (±0.58)	0.66 (±0.54)
*Eubacterium*	Firmicutes	0.53	0.61 (±0.29)	0.66 (±0.44)	0.31 (±0.23)

^**a**^shows taxa which represent >0.5% of the total reads in all samples combined.

*TM7_genera_incertae_sedis

**Fig 3:**
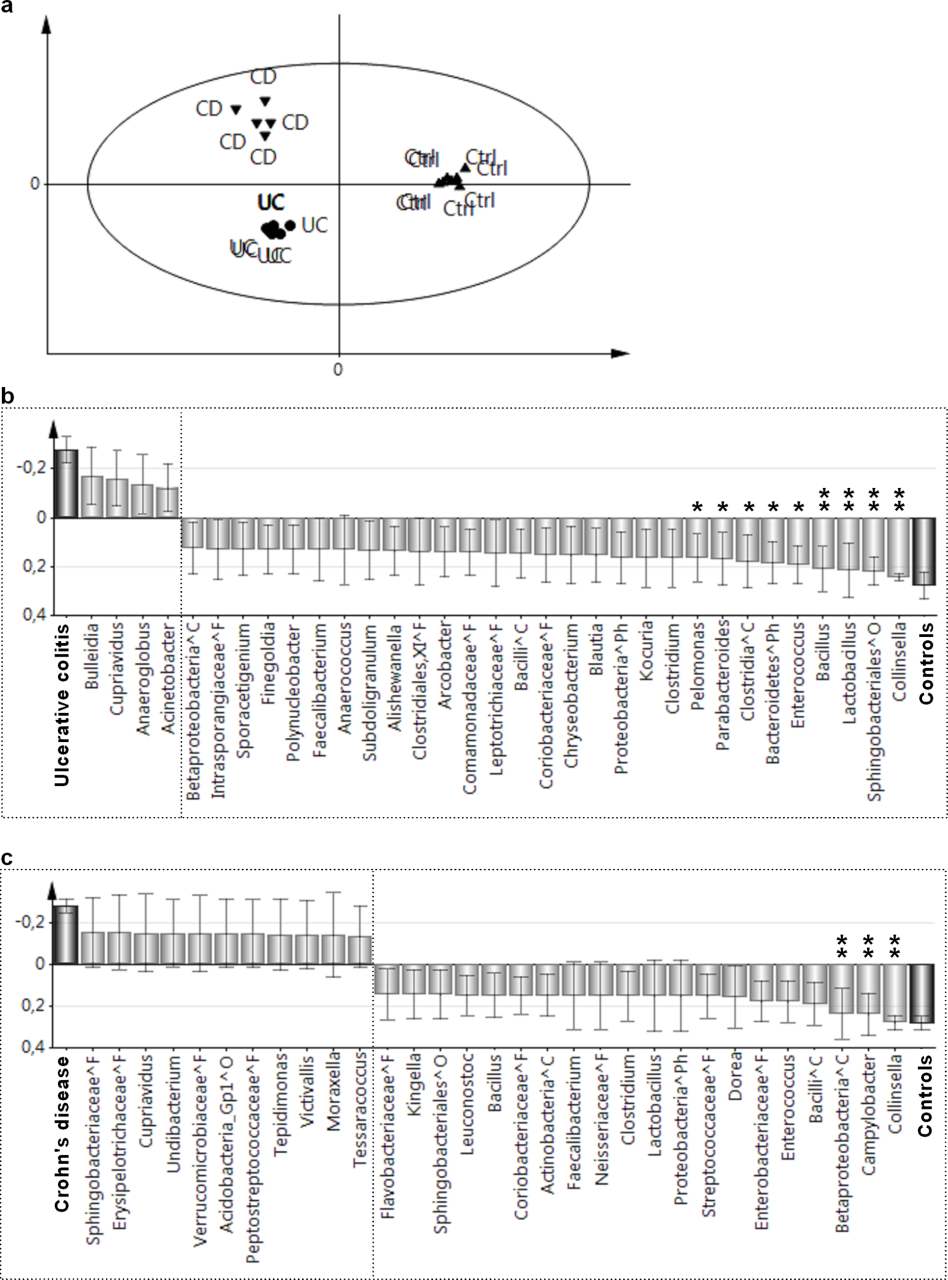
Differences in duodenal microbiota composition between children with ulcerative colitis or Crohn’s disease and controls. In each individual, the presence or absence is noted of each of the identified 322 OTUs, which are usually defined at the genus level. Distinguishing the three diagnostic groups is achieved using Orthogonal Partial Least Squares (OPLS). OTUs that contribute little to the separation (VIP value of <1.5) are eliminated (see *Patients and Methods*section). **A)** OPLS score plot. Each individual is represented by a symbol, the position of which is determined by the totality of the OTUs in the microbiota of that individual. Individuals in the three diagnostic groups: Crohn’s disease (CD, N = 5), ulcerative colitis (UC, N = 8), and symptomatic controls (Ctrl, N = 8) appear as separate clusters. **B)** Loading plots showing the OTUs that make the strongest contributions to distinguishing between patients with ulcerative colitis and controls **C)** Loading plots showing the OTUs that make the strongest contributions to the distinction between patients with Crohn’s disease and controls. Bars pointing in the same direction as the diagnosis bar are more common for the individuals with that diagnosis. OTUs that show the strongest contributions to the respective models were tested for differences in prevalence between patients and controls using Fisher’s exact tests; the levels of significance are indicated above the respective bars as follows: *, *P*<0.05; **, *P*<0.01. OTUs that could not be classified to the level of genus are denoted by the lowest taxonomic order to which they could be assigned using the following symbols: ^Ph, Phylum; ^C, Class; ^O, Order; and ^F, Family.

Differences in microbial composition were analyzed using the multivariate pattern recognition method OPLS, which investigates whether the presence or absence of each of the 322 OTUs in each patient would distinguish the three diagnostic groups. As shown in Fig 3A, patients with ulcerative colitis or Crohn’s disease could be readily separated from the controls based on the distribution patterns of the OTUs in their microbiota.

Fig 3B shows the OTUs that contributed the most towards distinguishing between the patients with ulcerative colitis and the controls. The bars that are pointing upwards in the figure represent OTUs that are more prevalent in children with ulcerative colitis, while the bars that are pointing downwards represent bacteria that are more prevalent in the controls; the larger the bar, the stronger is the contribution to the model. Univariate analyses (Fisher’s exact test) were performed to confirm unequal distribution of OTUs that contributed most to the multivariate model. Several taxa were found at lower frequencies in the patients with ulcerative colitis than in the controls (right-hand side of the diagram), including *Collinsella* (*P* = 0.001), *Lactobacillus* (*P* = 0.007), *Bacillus* (*P* = 0.007), and a sequence classified as belonging to the order *Sphingobacteriales* (*P* = 0.007). Other OTUs were also detected less frequently in the patients with ulcerative colitis, as compared to the controls (Fig 3B), albeit with moderate significance levels, so these differences should be interpreted with caution. Notably, few OTUs were more prevalent in the patients with ulcerative colitis than in the non-IBD controls (left-hand side of the diagram), and in no case were the differences statistically significant in the univariate analysis.

Fig 3C identifies the OTUs that contributed the most to differentiating the microbial compositions of the patients with Crohn’s disease and the controls. Consistent with the findings in patients with ulcerative colitis, the genus *Collinsella* was less prevalent among patients with Crohn’s disease than among the controls (*P* = 0.007). In addition, *Campylobacter* (*P* = 0.007) and an unidentified member of the class *Betaproteobacteria* (*P* = 0.007) were less prevalent in patients with Crohn’s disease than in the non-IBD controls.

**Fig 4:**
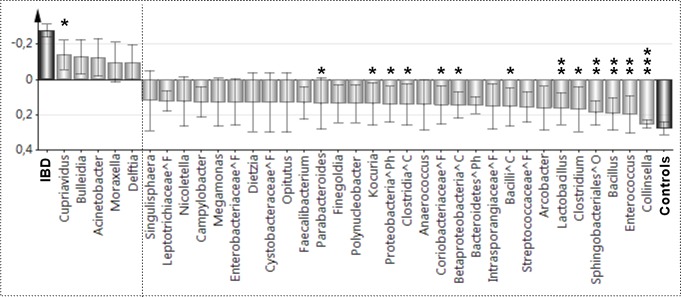
Differences in duodenal microbiota patterns between children with IBD and control children. Children with ulcerative colitis (N = 8) or Crohn’s disease (N = 5) were grouped together as IBD (N = 13) and compared with the control children (N = 8) regarding the presence or absence of each of the 322 OTUs identified in the entire data set, using OPLS. OTUs that contribute little to separating the groups were eliminated using the VIP approach (VIP value of <1.5, see *Patients and Methods*section). The bars that point upwards represent OTUs that are more prevalent in patients with IBD, while the bars that point downwards represent bacteria that are more prevalent in the controls; the larger the bar, the stronger is the contribution of those OTUs to the model. OTUs that showed the strongest contributions to the respective models were tested for differences in prevalence between patients and controls using Fisher’s exact tests; the levels of significance are indicated above the respective bars as follows: *, *P*<0.05; **, *P*<0.01; ***, *P*<0.001. OTUs that could not be classified to the level of genus are denoted by the lowest taxonomic order to which they could be assigned, using the following symbols: ^Ph, Phylum; ^C, Class; ^O, Order; and ^F, Family.

Fig 4 shows a comparison of the duodenal microbiota of children with IBD (i.e., children with ulcerative colitis and children with Crohn’s disease combined, N = 13) and the non-IBD controls (N = 8). The duodenal microbiota of children with IBD significantly less often contained OTUs that were identified as being from the genera *Collinsella* (*P*<0.001), *Enterococcus* (*P* = 0.003), *Bacillus* (*P* = 0.003) or *Lactobacillus* (*P* = 0.007), or to the order *Sphingobacteriales* (*P* = 0.003). Several other differences were found, although their levels of significance were moderate. This included an increased prevalence of *Cupriavidus* in patients with IBD compared with the controls (*P* = 0.046).

Table 2 shows the read abundance in each subject of 17 taxa that displayed significant differences between children with ulcerative colitis or Crohn’s disease and non-IBD controls (see Figs 3B, 3C and 4). The total number of reads per individual was 31k±12k (see Fig 2B).

**Table 2:** Number of reads of taxa that differed significantly in the OPLS analysis between the diagnostic groups.

Taxon	Number pf reads per sample																				
	Crohn's disease (n = 5)					Ulcerative colitis (n = 8)								Controls (n = 8)							
*Collinsella*	0	0	0	0	0	0	0	0	0	0	0	0	11	5	10	7	3	8	1	2	3
*Enterococcus*	0	0	0	0	0	0	0	0	0	0	0	0	0	4	12	2	0	8	11	0	0
*Bacillus*	0	0	0	65	0	0	0	0	0	0	0	0	0	0	2	14	0	1	2	6	2
*Sphingobacteriales*	0	0	0	9	0	0	0	0	0	0	0	0	0	0	5	17	16	5	5	0	6
*Clostridium*	0	0	0	0	0	0	0	0	0	0	0	0	0	2	0	4	13	0	0	0	4
*Lactobacillus*	26	0	0	24	20	0	1	0	0	0	20	0	0	3	81	17	18	7	18	1	14
Bacilli	0	0	4	0	0	0	3	0	0	1	0	0	37	2	1	18	9	22	0	2	9
Betaproteobacteria	0	30	0	0	0	0	0	9	7	3	3	0	10	2	12	6	32	8	8	3	1
*Coriobacteriaceae*	0	0	0	0	0	0	0	0	0	0	0	0	0	4	7	0	1	2	0	0	0
*Clostridia*	3	0	1	0	0	0	0	0	0	0	0	0	2	16	22	0	10	2	3	0	11
*Proteobacteria*	28	9	0	25	0	2	0	6	0	0	0	4	8	1	2	16	7	16	2	4	2
*Kocuria*	12	0	0	0	0	0	0	0	0	0	0	0	0	5	0	5	4	0	0	0	2
*Parabacteroides*	28	0	0	8	0	0	0	0	0	0	0	0	6	52	19	0	2	3	0	1	3
*Bacteroidetes*	5	0	5	63	0	0	0	3	0	0	0	0	20	4	65	8	8	14	13	0	9
*Pelomonas*	26	16	3	0	12	0	3	0	17	0	0	0	0	12	37	8	23	4	13	0	13
*Campylobacter*	22	0	0	0	0	313	189	16	242	289	0	59	51	231	74	48	6	39	1	2	4
*Cupriavidus*	38	0	0	8	0	0	3	0	11	0	1	2	0	0	0	0	0	0	0	0	0

## Discussion

In this pilot study, duodenal fluids were sampled from treatment-naïve children who were undergoing diagnostic work-up due to suspected IBD. The DNA samples extracted from the fluids were analyzed by pyrosequencing and, after a diagnosis had been established, the duodenal microbiota compositions were compared between children with IBD and non-IBD controls.

The children with ulcerative colitis were found to have an aberrant microbial composition, as compared to the microbiota of the non-IBD controls. A major finding was that the duodenal microbiota of the children with ulcerative colitis displayed significantly lower richness, i.e., contained fewer bacterial taxa (OTUs) than the microbiota of non-IBD control children. The low level of richness was seen across three major phyla: *Actinobacteria*, *Bacteroidetes*, and *Firmicutes*, all of which were represented by significantly fewer OTUs in the children with ulcerative colitis compared with non-IBD controls. The low microbial richness was not due to few bacterial cells in the duodenal sample, as equal numbers of sequencing reads were generated from the samples derived from the children with IBD and the control children. Although the microbiota of children with Crohn’s disease tended to contain fewer OTUs than those of the controls, there was greater variability in this group and the difference did not reach statistical significance. In this context, several studies have shown reduced complexity of the large bowel microbiota in children with ulcerative colitis [27] and in children with Crohn’s disease [28, 29], as compared to controls. Thus, the observed reduction in microbial complexity in both the small and large intestines may well have a common cause. One such cause may be reduced exposure to microbes, e.g., *via* the food and/or environment.

To our knowledge, this is the first study that has examined the proximal small intestinal microbiota in patients with IBD. As there have been no previous studies of the duodenal microbiota in patients with IBD, comparisons are difficult. In dogs with spontaneous IBD, duodenal biopsies were found to contain fewer species than those collected from healthy dogs, and the percentages of *Fusobacteria*, *Bacteroidaceae*, *Prevotellaceae*, and *Clostridiales* were lower. Instead, their duodenal microbiota contained more members of the Proteobacteria phylum, e.g., *Enterobacteriaceae*, *Diaphorobacter* and *Acinetobacter* [30, 31]. The microbiota of the terminal ileum has been investigated in various studies [14, 15], from which it has been reported that the microbiota of ileal biopsies from children with Crohn´s disease have an increased prevalence of several obligate anaerobes, such as *Faecalibacterium prausnitzii*, as compared to the microbiota of ileal biopsies from controls. However, these findings are not necessarily in discordance with the results of the present study, since the terminal ileum microbiota is more akin to the colonic microbiota, with the duodenal microbiota bearing a closer resemblance to the oral/respiratory microbiota [32, 33]. Thus, the Proteobacteria of the colon foremost comprise the *Enterobacteriaceae* family, while in the upper small intestine, *Haemophilus* and *Neisseria* are the most common members of the Proteobacteria phylum. Although a recent study demonstrated a decrease in *Streptococcus* (phylum Firmicutes), *Haemophilus* and *Neisseria* (phylum Proteobacteria) in saliva [34], we observed no reduced abundance of these genera in the duodenal microbiota in IBD patients.

Another notable finding is that the children with IBD had far fewer reads of unknown origin than the non-IBD controls. In the non-IBD controls, 3.5% of the sequences could be classified as Bacteria and an additional 3.9% could not even be classified as Bacteria, while the corresponding percentages for children with IBD were at least nine-times lower. These two measures were highly correlated, i.e. a sample with large numbers of sequences classified only as Bacteria also had large numbers of sequences that remained unclassified. It should be noted that 16S rRNA gene sequencing has limitations, in that “false species” may be deduced due to sequencing artifacts, the region of the 16S rRNA gene being sequenced, chimera formation, and the lack of uniform reliability of the underlying taxonomic scheme [35]. However, we did remove all singletons as well as potential chimeric sequences, and only used high-quality reads with a score ≥25. Furthermore, as pointed out above, the total numbers of reads were similar in the three groups. Thus, the findings were not attributable to overall lower yields of DNA from the patients with IBD. As we used quite long reads (500 nt on average), the possibility of falsely classifying reads to known genera may be lower than if shorter reads are used. Thus, we tentatively regard the finding of quite high levels of unclassifiable reads in several of the control children, but low levels of unclassifiable reads in the IBD groups as support for the notion of low microbial breadth in children with IBD. Bacterial genera which differ significantly in the OPLS analysis have abundances below 0.5% of the total reads (Table 2). Although they are present in fairly low amounts, we do not believe that the signals are false. These OTUs were detected in almost all control samples but only found in very few patients with IBD. It is unlikely that this pattern would result from sequencing errors, but that it instead reflects the less rich microbiota of the IBD patients, particularly those with ulcerative colitis.

The observed reductions in the richness and breadth of the small intestinal microbiota may reflect a more restricted microbial exposure in children with IBD, as compared to the controls. This idea is compatible with the previous findings that IBD occurs more frequently in areas of the world that have good levels of hygiene [36], and that both early infections [37] and growing up in a farming environment [38] are partially protective against IBD. The small intestine is crucial for the regulation of systemic and mucosal immunity, and tolerance to microbes and soluble antigens to which we are exposed *via* the gut [16]. Antigen-specific mucosal immune responses are induced in the Peyer’s patches located in the small intestine, from where activated lymphocytes seed the small and large intestinal mucosa as effector cells. The small intestine is also the site of induction of oral tolerance to harmless antigens [16]. The commensal microbiota affects immune function, since germfree animals have poorly developed Peyer’s patches [39], reduced capacity to develop oral tolerance [40], and impaired functional activities of the regulatory T cells [41], as compared to conventionally reared animals. Therefore, exposure of the mucosal immune system to a broad variety of bacteria may confer a broad immunostimulation that acts through as yet unknown mechanisms to promote healthy immune regulation, while a microbiota of low complexity may not afford the sufficient immune stimuli. Accordingly, a complex commensal gut microbiota in the neonatal period is protective against the development of allergy, which is another disorder of immune regulation [42].

However, several other factors than exposure could explain changes in the microbial community. Reduced microbial complexity in patients may be due to an altered intestinal milieu, e.g. local inflammation. However, in the present study, ulcerative colitis was associated with the clearest alterations in duodenal microbiota composition, despite the fact that in this disease, the inflammation primarily affects the colon. Still, epithelial barrier, and permeability defects have been detected in the duodenal biopsies from patients with IBD [43, 44], along with markers of local inflammation that were not reflected in general disease activity [44, 45]. Furthermore, although we excluded all children whose diet had been modified due to the disease, as well as those treated with antacids, antibiotics or other medication, bacterial richness might be influenced by transit time, anemia and overall lower food intake that may reduce acid and bile secretion. However, the fact that the total bacterial population levels, measured by the total number of reads, were not reduced in the children with IBD, speaks against a more hostile environment in the duodenum of patients with IBD.

A weakness of the present study is the lack of healthy controls. It is not considered ethically justifiable to perform invasive procedures, such as endoscopy, on healthy children. As a result, few studies have examined the composition of the small intestinal microbiota in healthy children. However, the microbiota of the non-IBD group resembled that reported previously as being dominated by the *Firmicutes* and *Proteobacteria* phyla and the *Streptococcus* and *Neisseria* genera [18, 19]. Furthermore, a recent study indicates that the small intestinal microbiota is very similar in patients with IBS and healthy controls [46]. Another limitation of the present pilot study is the low number of participating children, especially those with Crohn’s disease. Therefore, the results should be interpreted with caution and need to be verified in a larger study. The strengths of the study include well characterized treatment-naïve subjects with new onset of disease and the use of a sequencing methodology with appropriate read length (~500 nt) and sequence depth (~30,000 reads/individual). Considering that the microbiota of the duodenum is much less dense than that of the colon (~10^4^ CFU/g vs. 10^11^ CFU/g).

In the pyrosequencing analysis of the duodenal microbiota samples, we attempted to assign each sequence to a genus, which can be achieved with 90% accuracy when analyzing 400-nt segments [47]. We found a reduced presence of certain bacterial genera in the children with ulcerative colitis, among which *Collinsella* was also reduced in the children with Crohn’s disease, as compared with the controls. The genus *Collinsella* belongs to the Actinobacteria phylum and comprises four species, of which *C*. *aerofaciens* is a common member of the human large bowel microbiota [48]. *C*. *aerofaciens* possesses bile salt hydrolase activity [49], and isolates from chicken intestines have been shown to be able to de-toxify mycotoxins [50]. Notably, commensal colonization by *Collinsella* is lower in Western populations, as compared to populations in developing countries [51], and in populations that are at high risk for developing colon cancer, as compared with controls [52]. In addition, *Collinsella* is less prevalent in the relatives of patients with Crohn’s disease [53], and in patients with inflammatory bowel syndrome [54].

Other genera that were found at reduced prevalence in the duodenal microbiota of children with ulcerative colitis included *Lactobacillus* and *Bacillus*. Lactobacilli are commonly detected in the oral and intestinal microbiota [55] and are also widely found in food products. Probiotic treatment with *Lactobacillus reuteri* increases the serum levels of both conjugated and unconjugated bile acids [56]. *Bacillus* is an aerobic spore-former, and its spores are ubiquitous in the environment and in foodstuffs. Furthermore, an OTU belonging to the *Sphingobacteriales* order (phylum *Bacteroidetes*), which could not be assigned to a genus, was also detected less frequently in patients with ulcerative colitis, as compared to the controls. The name *Sphingobacteriales* alludes to the production of sphingolipids. Sphingolipids are recognized by invariant natural killer T (iNKT) cells [57] and exert a protective effect against oxazolone-induced colitis [58], which is mediated by iNKT cells. Sphingolipids could also play an intermediary role in the crosstalk between intestinal immunity and microorganisms [59].

We found fewer changes in the duodenal microbiota of patients with Crohn’s disease compared with the controls, which may reflect the lower number of patients in this group. In addition to *Collinsella*, as mentioned above, *Campylobacter* and an unclassified member of *Betaproteobacteria* were found less frequently in the patients with Crohn’s disease than in the controls. The *Campylobacter* genus contains 24 species, of which only two, *C*. *jejuni* and *C*. *coli*, are recognized pathogens. Little is known about the ecologic effects of other *Campylobacter* species. A recent study used microaerophilic cultivation and PCR screening to detect specifically *Campylobacter* and other Gram-negative facultative bacteria in colonic biopsies collected from children with newly diagnosed IBD and controls [60]. In that study, various *Campylobacter* species other than *C*. *jejuni* and *C*. *coli* were commonly detected in both the patients with IBD and the controls, and no association with IBD was found [60].

In summary, this pilot study demonstrates an aberrant duodenal microbiota, which is characterized by low richness and a reduced prevalence of certain bacterial genera, in children with newly diagnosed ulcerative colitis. Thus, microbiota dysbiosis in ulcerative colitis is not confined to the colon, which is the main site of the gut inflammation.

## Supporting information

S1 Table Number of sequences per operational taxonomic unit. The raw sequences were analyzed using the software package Quantitative Insights into Microbial Ecology (QIIME), and reads with a quality score of ≥25 and a read length of 200–500 nucleotides were retained for analysis. Operational Taxonomic Units (OTUs) were chosen based on repetitive sequences derived from sequence clustering using the UCLUST software, with a minimum pairwise identity of 97%. A representative sequence for each OTU was chosen based on abundance and length, aligned using the PyNAST software (QIIME), and assigned to the genus level (when possible) using the Ribosomal Database Project (RDP) classifier nomenclature.
(XLSX)

S2 Table Number of reads per phylum for the patients. (XLSX)

S1 Fig **Pearson’s correlation coefficient analyses** between “Bacteria only” and “unclassified reads” for: a) patients with Crohn’s disease; b) patients with ulcerative colitis; and c) Controls.
(DOCX)
