# Supplementary material for: Low-complexity microbiota in the duodenum of children with newly diagnosed ulcerative colitis
Source: PLoS One. 2017 Oct 19;12(10):e0186178. doi: 10.1371/journal.pone.0186178 (PMC5648149; doi:10.1371/journal.pone.0186178)
Supplement: S1 Fig — Pearson’s correlation coefficient analyses between “Bacteria only” and “unclassified reads” for: a) patients with Crohn’s disease; b) patients with ulcerative colitis; and c) Controls. (DOCX) [file pone.0186178.s003.docx]

S1 Figure.
